# Psychological Outcome in Young Survivors of Severe TBI: A Cross-Informant Comparison

**DOI:** 10.1155/2015/406057

**Published:** 2015-10-15

**Authors:** Karoline Doser, Ingrid Poulsen, Anne Norup

**Affiliations:** ^1^Research Unit on BRain Injury Rehabilitation Copenhagen (RUBRIC), Clinic of Neurorehabilitation, Traumatic Brain Injury Unit, Rigshospitalet, 2650 Copenhagen, Denmark; ^2^Department for Clinical Pedagogic and Therapeutic Studies, Catholic University of Applied Sciences Freiburg, 79104 Freiburg, Germany; ^3^Department of Neurology, Rigshospitalet, 2600 Copenhagen, Denmark

## Abstract

*Objective*. To investigate the psychological outcome and the agreement between self-ratings and proxy-ratings in young individuals after severe traumatic brain injury (TBI). *Methods*. Twenty pairs of former patients who sustained a severe TBI in their adolescence or early adulthood and their significant others (SOs) were contacted around 66 months after injury to complete a measure of psychological and behavioral problems. The Adult Self-Report 18–59 and the Adult Behavior Checklist 18–59 were used. *Results*. Results showed significant differences compared to the normative sample in the domains withdrawal, attention, and intrusive and internalizing problems. Good or excellent levels of agreement were found between the self-rating and the proxy-rating in overt areas such as somatic complaints and aggressive and intrusive behavior. Fair or poor levels of agreement were found in nonovert areas such as anxiety and depression, withdrawal, thought and attention problems, and personal strength. *Conclusion*. The findings show that young patients experience psychological dysfunction. Our study suggests that the use of either a self-rating or a proxy-rating would be appropriate for evaluating overt domains, regarding the good to excellent levels of agreement. However, in nonovert domains, such as withdrawal and attention, an additional proxy-rating from a SO could provide supplementary information and build a more complete objective assessment.

## 1. Introduction

Traumatic brain injury (TBI), defined as damage of brain tissue caused by external mechanical force, is the foremost cause of acquired disabilities among young adults [1]. TBIs are also known as one of the leading causes of death [2], where adolescents, young adults, and the elderly are at the highest risk [3, 4].

After sustaining a severe TBI, impairments including physical, cognitive, emotional, psychosocial, and behavioral limitations are common [5–7]. Following the acute state, residual motor and sensory deficits may persist in a wide range [8]. These limitations have a significant short- and long-term impact on social, neuropsychological, behavioral, and academic domains for the majority of TBI victims [9, 10]. Adolescents are especially reported to be more susceptible to long-term disability due to the vulnerability of the immature brain [11–16]. Consequently, young individuals have exhibited residual impairments in a range of areas including emotional, behavioral, adaptive, and cognitive dysfunction [14, 17]. Adolescence is defined as a period of transition from being a dependent child to an independent and a self-determined adult. A TBI within this time frame not only causes physical and cognitive impairments, but also interrupts the natural process of maturation. This interruption subsequently impairs still-developing skills and results in psychological difficulties [18, 19]. These psychological difficulties, such as depression, are significant, since they impact the quality of life for adolescents surviving TBI [20].

To assess the psychological outcome, standardized measures are commonly used. This can be done in two ways: asking the patient for his or her subjective opinion by using a self-rating scale or asking a significant other (SO) to describe the patient by using a proxy-rating scale. For populations with cognitive disability, such as after TBI, some people may be unable to provide an accurate self-report due to impaired cognition or communication. Seel et al. [21] reported that people with very severe injuries tended to underrate their problems, whereas people with mild injuries typically rated problems as occurring more frequently than family members' reports. However, very often, either a self-rating scale or the proxy-rating scale is utilized. On the one hand, it can be difficult for patients to subjectively reflect and describe their perceived psychological impairments. On the other hand, challenges with proxy reports occur when specific measures of outcome require knowledge about unobservable, internal experiences [22]. Obtaining both a subjective opinion from the patient and a proxy opinion could lead to a more trustworthy evaluation. Proxy opinions can be obtained by a SO completing a cross-informant rating system.

Cross-informant ratings can be beneficial, for instance, when awkward social areas are assessed in which individuals tend to provide inaccurate but desirable answers due to youth and insecurities. Cross-informant rating systems are available in a variety of instruments and have been used in prior research [21–25]. However, it is important to consider that using different people to assess psychological functioning can provoke various results.

The level of agreement between the self-rating score of a patient and the proxy-rating score of a SO (parent, sibling, partner, or primary caregiver) has been used by researchers in the past to investigate psychological outcomes following a TBI. Also, by studying the agreement between patient and proxy reported scores, researchers have been able to examine a patient's level of self-awareness [22]. Impaired self-awareness is a common problem after TBI and is often associated with a decreased functional outcome and poor compliance with rehabilitation [26]. Impaired self-awareness can lead to over- or underestimation of the patient's own condition. A high level of agreement between self-rating and proxy-rating reports can, in turn, reflect a high level of self-awareness in a patient.

Previous research also suggests that the exclusive use of proxy responses should be used with caution when evaluating a patient's participation level in social activities. Researchers found that proxy responses could be biased and overly critical in evaluating the patient's social participation [24]. Therefore, independent proxy data can be acceptable for research purposes. However, in order to assess outcomes and set future clinical and rehabilitation goals, input from both sides, persons with TBIs and their SOs, should be considered [25].

The agreement between patient and proxy perceptions of psychosocial outcomes was previously assessed in an Australian study using the ASR 18–59 and the ABCL 18–59 on 33 individuals and their SOs 16 years after mild, moderate, or severe pediatric TBI (mean age at injury *M* = 4.70, SD = 1.74) [23]. The results showed a generally poor to fair agreement on most of the investigated subscales of internalizing and good to excellent agreement on communication and drug abuse.

Green et al. (2012) found there to be an excellent agreement between adolescents and their parents in the assessment of the psychological outcomes using the Sydney Psychosocial Reintegration Scale for Children (SPRS-C) after a childhood TBI (age at injury ranged from 0 to 5 years). However, there was less of an agreement regarding quality of life [22]. It is important to bear in mind, however, that quality of life is a subjective perception, which is very difficult to describe from an external perspective.

The main focus in previous studies has been either adulthood or early childhood. Apart from the small number of studies addressing pediatric patients after TBI, no study was identified as specifically addressing patients who sustained a severe TBI during their adolescence or early adulthood.

Most research has described consequences based on information from either a self-rating or a proxy-rating system.

Researchers generally agree that the perceptions of impairments and difficulties are most likely to differ between a self-reporting perspective and the perspective of a family member or caregiver [21–25].

Recent research reports mostly on mixed samples consisting of mild, moderate, and severe TBI. These studies have very small numbers of participants with severe TBIs [22, 23]. In future research, gaining more knowledge regarding the perception of impairments and difficulties of people with TBIs would lead to a better understanding of how the involvement of a SO would be beneficial in the identification of psychological problems.

## 2. Objective

The aim of this study was twofold. First, the study intended to investigate the psychological functioning of individuals after sustaining a TBI in their adolescence or early adulthood and compare the results to a normative sample. Second, the study investigated the agreement between the self-ratings by the young adults and the cross-informant ratings by the SOs.

Based on previous research, the expectation was to find higher levels of psychological dysfunction in the TBI sample than in the normative sample. The authors also expected to find a lower agreement between patient reporting systems and their SOs within internalizing domains and a higher agreement in externalizing domains. This paper reports the results of a study investigating the psychological outcome in young patients after severe TBI. The first part of the study investigated in which domains and at which level the young patients perceived difficulties, and the second part of the study addresses the agreement between the patients and their SOs and is reported in the current paper.

## 3. Methods

The patients included in the study had all been admitted to a subacute neurorehabilitation department between the years 2000 and 2013 and were contacted during 2014 for a follow-up assessment in the chronic phase after injury. The department has an admitting area covering the eastern part of Denmark and provides subacute inpatient rehabilitation and follow-up visits to patients, who sustain severe brain injuries.

### 3.1. Inclusion Criteria

Inclusion criteria were as follows:

1. Patients who sustained a TBI.
2. Glasgow Coma Scale (GCS) score under 9 at time of injury, indicating a severe TBI.
3. Between the ages of 14 and 21 at time of injury and at the age of 18 or older at the follow-up assessment.
4. Level of cognitive functioning, score 7 and 8 on the Rancho Los Amigos scale at discharge from subacute inpatient rehabilitation.
5. Living in Denmark, within the admitting area.

### 3.2. Exclusion Criteria

Exclusion criteria were as follows:

1. Living outside the admitting area, that is, Greenland and the Faroe Islands (part of Denmark).
2. Not Danish speaking.

### 3.3. Participants and Procedure

**Figure 1:**
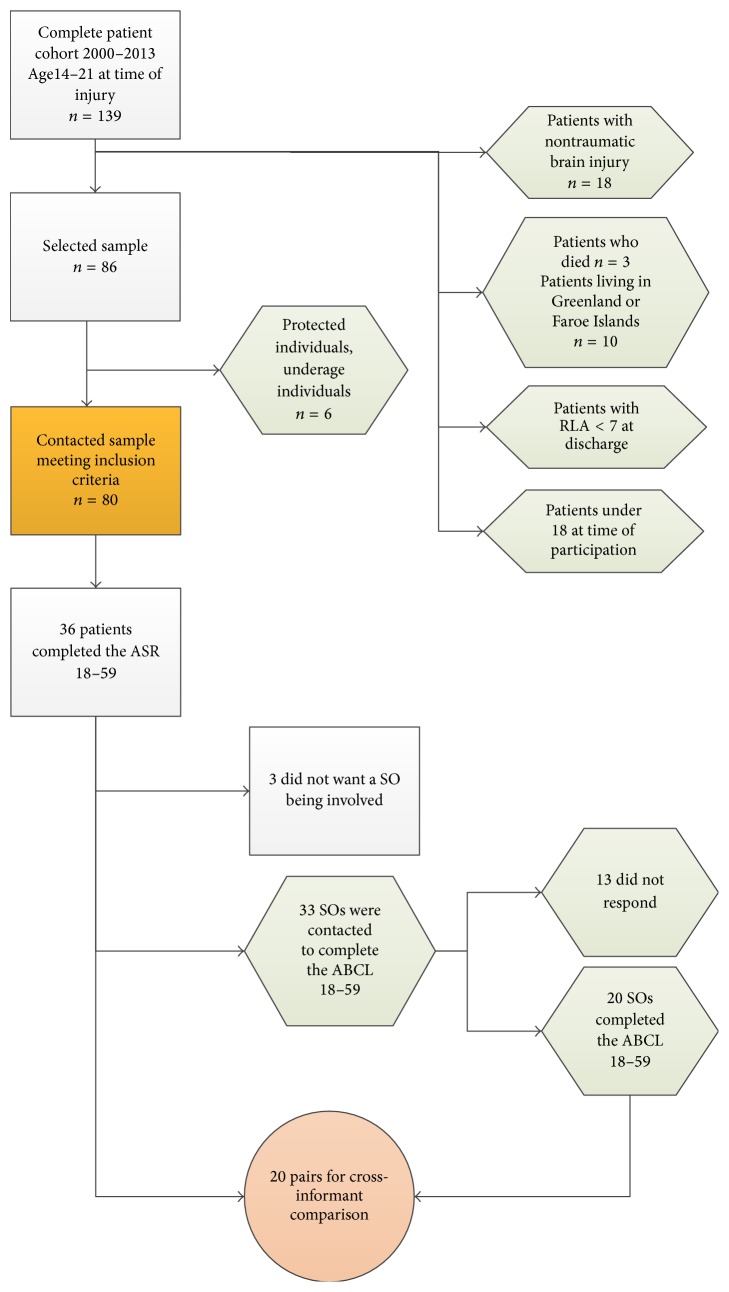
Flowchart of participant enrolment.

Eighty former patients, who were admitted for subacute rehabilitation at the clinic of neurorehabilitation, between the ages of 14;1 years to 21;11 years were invited to participate in a questionnaire during the chronic phase after injury. Thirty-six completed and returned the questionnaires and 33 provided written consent to contact a SO, such as a parent, caregiver, or spouse, for a cross-informant rating of the measure. Of the contacted SOs, 20 completed and returned the sent materials (Figure 1). The mean time since injury was 66.1 months (SD = 46.6 months).  Table 1 displays the sample characteristics.

The study was conducted according to the Regional Ethical Committee, the National Board of Health, and approved by the Danish Data Protection Agency (J. number 2007-58-0015).

### 3.4. Measures

To describe the severity of injury and the patient's condition, three indicators were used: the length of posttraumatic amnesia (PTA), the Rancho Los Amigos (RLA) score [27], and the length of stay (LOS) at the rehabilitation unit.

The mean length of posttraumatic amnesia (PTA) was assessed prospectively using the Galveston Orientation and Amnesia Test (GOAT) [28] by neuropsychologists. This test was used as a standard assessment during hospital rehabilitation. After scoring at least 75 points on two consecutive days, the PTA is considered as terminated.

The patient's level of consciousness was assessed using the Rancho Los Amigos (RLA) scale [27]. The scores range from Level 1, which describes a comatose condition with no observable response, to Level 8, which is a condition with purposeful and appropriate responses [27]. An RLA score of 7 (automatic, appropriate response) or 8 (purposeful, appropriate response) was chosen as inclusion criteria, to ensure that the contacted patients regained the ability to complete the study's measures.

The ASEBA material, developed by Achenbach and Rescorla [29], provides several age-related instruments to assess the psychological functioning of individuals in the form of a self-rating system as well as corresponding cross-informant rating system. The Adult Self-Report (ASR 18–59) and the Adult Behavior Checklist (ABCL 18–59) acted as the primary outcome measures of the study. These questionnaires assess competencies and adaptive functioning as well as behavioral, emotional, and social problems. The questionnaires consist of 123 closed and three open items as well as a set of questions regarding the living status and demographic information. Each item represents a statement scored by a Likert scale where 0 indicated “not true,” 1 “somewhat or sometimes true,” and 2 “very true or often true.” The items are distributed between the Adaptive Functioning Scales* ((1) friends, (2) spouse/partner, (3) family, (4) job, (5) education, and (6) personal strength)*, the syndrome scales* ((1) anxiety/depression, (2) withdrawal, (3) somatic complaints, (4) thought problems, (5) attention problems, (6) aggressive behavior, (7) rule-breaking behavior, and (8) intrusive behavior)*, the combined scales* ((1) internalizing, (2) externalizing, and (3) critical items)*, the DSM-orientated scales* ((1) depressive problems, (2) anxiety problems, (3) somatic problems, (4) avoidant personality problems, (5) attention deficit/hyperactivity problems, and (6) antisocial personality problems)*, and the Substance Use Scales* ((1) tobacco, (2) alcohol, and (3) drugs)*. The scores of the 126 items of the ASR 18–59 are allocated to the specific subscales. Raw scores, *t*-scores, and percentiles are calculated by the software or via hand-scoring for each scale and are presented in a profile. The *t*-scores describe whether the score of a subscale is within the normal range or in a border range or clinically significant. After scoring, a profile displays the levels of each subscale as normal, critical, or clinical range [30]. By using Pearson correlations, the reliability of this measurement was reported as generally very high, with “all test-retest* r*s being significant at *p* < 0.01 and most being in the 0.80s and 0.90s” [31].

For the present study, only *t*-scores of the syndrome scales as well as the combined scales for substance abuse, internalization, externalization behavior, critical items, and adaptive functioning were used to enhance interpretation. For the syndrome scales (clinical range > 64) and combined scales (clinical range > 61), a higher *t*-score indicates more problems, as opposed to the Adaptive Functioning Scales, where a lower *t*-score (clinical range < 32) indicates worse functioning.

### 3.5. Analysis

All data was entered into and screened for violations of normality and analyzed by SPSS version 19.00.

#### 3.5.1. Demographic Data

To characterize the study participants, demographic information as well as injury related information was obtained. Patient demographic information consisted of gender, age at the time of injury and participation, and the time since the original injury occurred. The relationship between the patient and the SO as well as the cohabitation status was recorded for each pair. Measures of central tendencies, such as the mean, were calculated. Measures of variability, including standard deviation (SD), were also applied. All analyses were two-tailed and used a significance level of *p* < 0.05 (if not noted otherwise).

#### 3.5.2. ASR 18–59 and ABCL 18–59

Means were computed based on the *t*-scores for the syndrome subscales as well as for the combined scales.

To investigate whether the values of the self-rating and the proxy-rating were significantly different to the normative sample, one-sample *t*-tests were performed. Therefore, the whole responding sample of the young patients (*n* = 36) (see Figure 1) and the responding SOs (*n* = 20) were compared with the normative sample from Achenbach and Rescorla (2003) using one-sample *t*-tests. Normative data for the age range 18–35 was used.

### 3.6. Cross-Informant Comparison

Each subscale's self-rating scores were calculated using the ASR 18–59, whereas the ABCL 18–59 was used to calculate the proxy-rating scores. Intraclass correlations (ICC) were used to test whether or not the self- and proxy-rating answers were consistent. The suggested levels of clinical significance were used to rate correlations: ICC < 0.40 indicates a poor correlation, an ICC of 0.40 to 0.59 a fair level, an ICC of 0.60 to 0.74 a good level, and an ICC of 0.75–1.00 an excellent level [32].

### 3.7. Supplementary Analysis

In order to compare the LOS and the PTA between the responding and nonresponding participants, independent *t*-tests were applied. Spearman correlations were carried out to investigate whether a relationship existed between the time since original injury and the patient's level of psychological functioning.

## 4. Results

### 4.1. Demographics and Status

#### 4.1.1. Patients

The mean age of patients at the time of injury was 18.1 years (SD = 1.9) and 80% were male. The mean age at participation was 23.75 (SD = 4.9), and the mean time since injury was 66.1 months (SD = 46.6) with a range between 5 and 150 months. The mean PTA duration was 49.4 days (SD = 34.8) and the mean LOS at the TBI unit was 70.5 days (SD = 44.8) (see Table 2).

#### 4.1.2. Characteristics of the SOs

The majority of the SOs were parents (*n* = 18), except one who was a spouse and one who was a professional caregiver.

### 4.2. Psychological Outcome

Significant differences between the self-rating scores of the young patients and the normative sample were found in the subscales of withdrawal (*t*(35) = 2.63, df = 35, *p* = 0.013), attention (*t*(35) = 2.81, df = 35, *p* = 0.008), and internalization (*t*(35) = 2.25, df = 35, *p* = 0.031), all of which showed higher mean problem scores for the patients. Significantly lower patient reported scores were found in the subscale for intrusive behavior (*t*(35) = −2.06, df = 35, *p* = 0.046). No significant differences were found in the remaining subscales. The subscale of attention was the only category to result in significantly higher proxy-rating scores when compared to the normative sample (*t*(20) = 3.00, df = 19, *p* = 0.007) (Table 3).

### 4.3. Comparison between Self-Rating and Proxy-Rating

The scores of each subscale of the ASR 18–59 and the ABCL 18–59 were compared using paired two-sample *t*-tests. No significant differences were found between the mean ratings in the tested subscales (Table 4). The interrater agreement, assessed by using intraclass correlation (ICC), showed an excellent level of agreement within the subscales of somatic complaints. A good level of agreement was found within the aggressive and intrusive behavior of the syndrome scales, as well as a good level of agreement within the internalizing and critical items of the combined scales.

A fair level of agreement was found in the syndrome scales of anxiety and depression, attention problems, and rule-breaking behavior, as well as in the combined scales of externalizing and total problems. Finally, a poor level of agreement was found in the subscales of thought problems and personal strength (Table 4).

### 4.4. Supplementary Analyses

A set of supplementary analyses were conducted to investigate the effect that time since the original injury would have on the psychological functioning. Analyses were also run to compare responders' versus nonresponders' level of injury which might influence their ability to reply.

Spearman correlations were carried out, and a significant negative correlation was found only between the time since injury and the subscales of anxiety and depression (*r*s = −0.388, *p* = 0.019).

We compared duration of PTA and the age and the ratio of male and female in the group of subjects who responded to the participation invitation against those subjects who did not respond to the participation invitation during our follow-up. Our results showed that there was no significant difference between the PTA, age at injury, and age at participation. Only slight differences in the ratio of male and female were found (Table 5). Consequently, there was no indication that the nonresponders had more severe injuries than the group who completed the study material.

## 5. Discussion

This study investigated problems that are perceived by young individuals with severe TBI and their SOs by using a cross-informant comparison of psychological functioning. Our findings suggest that young patients perceive themselves to have significantly more psychological problems when compared to the normative sample. The comparison between the self-rating and the proxy-rating scores demonstrated good and excellent agreement in overt domains and fair to poor agreement in nonovert domains.

### 5.1. Psychological Outcome

Significant differences between the self-rating scores and the normative sample were identified. The participants expressed that they experienced withdrawal, attention, and internalizing problems, with means for these domains being significantly higher. Patients also reported a significantly lower mean of intrusive behavior when compared to the normative sample. The findings also indicate that the young patients perceive more psychological difficulties than their SOs detect because the SOs only reported significantly higher scores in one subscale, attention, when compared to the normative sample. This discrepancy may suggest a risk for the young former patients who experience problems but do not notify their closest relative of the intensity of their experiences. The differences in the ratings could also be due to overestimation of the problems by the young patients, as they might be accustomed to reporting their impairments and difficulties after injury, whereas parents may tend to underestimate and normalize the situation.

### 5.2. Agreement

An excellent level of agreement between former patients and their SOs in the somatic complaints subscale is not surprising, as physical impairments are more likely to be overt and more noticeable by the SOs. Patients are more likely to communicate somatic problems, since these difficulties can be named specifically, such as pain or physical discomfort. The good agreement in aggressive behavior subscale was also expected, as these issues might directly affect the SOs. A fair level of agreement was found in subscales addressing nonovert and passive behavior of individuals such as withdrawal and attention problems. Poor agreement was found in the subscales of thought problems and personal strength.

The proxy-rating scores for the categories of thought problems and personal strength were high when compared to the patients' corresponding self-rating scores. The lower agreement between SOs and patients supports the conclusion that these two domains are nonovert and therefore less visible to SOs. Consequently, it might be difficult for SOs to estimate the intensity of problems experienced by patients in the two nonovert domains of thought problems and personal strength. These results are also consistent with Hart et al. (2003), who stated that questions about physical abilities tend to yield more agreement than those regarding emotional or cognitive status [24, 33].

Within the combined scales, which consisted of several subscales, internalizing demonstrated a better agreement (good) then externalizing (fair). This was in contrast to our expectations, since internalizing behaviors are attributed to one's self and are often more difficult to observe than externalizing behavior such as aggression or substance abuse [33]. However, the symptoms of internalizing problems, such as depression, are known in society and are therefore more easily recognized when compared to externalizing behavior such as rule-breaking behavior.

Our study's findings also demonstrated a difference between self-reported and proxy reported mean scores in the combined scale of externalizing behavior. The proxy-rating scores showed an overall higher mean value than the patient's self-rating scores for externalizing behaviors. This finding suggests that the SOs identify more problems in this domain than the former patients are aware of.

It was also surprising that our study found a poor agreement between self-rating and proxy-rating results for the subscale of personal strength. The items used to measure personal strength encompassed the social environment of the former patients in domains such as friends, job, education, spouse, and family. This disagreement may be explained by decreased SO insight within those domains (e.g., job) but also by impaired self-awareness of the patient, which is common in TBI survivors.

An investigation of the agreement between outcome measures, as reported by proxy-rating and self-ratings in the chronic phase after TBI, has previously been studied by Rosema et al. (2014) using the ASEBA measures [23]. Rosema et al. conducted a study on 33 patients after mild, moderate, and severe childhood TBI (mean age 4.70 years, SD = 1.7 years) around 16 years after injury. An overall higher level of agreement was found within our current sample compared to their study. In Rosema et al.'s findings, an excellent agreement between the former patients and their SOs was only found in the scale of substance abuse. A fair agreement was noted in somatic complaints, externalizing, and total problem measurements. Finally, a poor agreement was documented in anxiety and depression, withdrawal, thought problems, attention problems, aggressive behavior, rule-breaking behavior, and internalizing. In conclusion, Rosema et al.'s findings showed a similar poor agreement for the overall internalizing symptoms; however, the difference between our results and Rosema et al. might very well be caused by the severity of injury received by the former patients. During our study, only severe cases were included; therefore, the level of support and the need for caregiving may have been higher. Consequently, the patients in our study might be more attached to their parents and share more of their daily life with them. This closer connection could have led to the parents possessing a better insight into the patient's current situation. Additionally, in Rosema et al.'s study, the research follow-up occurred after a longer period of time following the initial injury. Therefore, the patients may have gained more insight and therapy regarding their difficulties during this extended time following their injury. Finally, another reason for the higher agreement in our study may be explained by an older age at the time of injury. Varni et al. showed a trend toward higher intercorrelations with an increase in age. This phenomenon could possibly be explained by the greater verbal communication skills that are typically manifested with increased developmental age [34].

Using the Sydney Psychosocial Reintegration Scale for Children (SPRS-C) and the Pediatric Quality-of-Life Inventory 13–16 years following injury, Green et al. (2012) found excellent agreements in parent-adolescents ratings regarding the psychosocial outcome and quality of life after childhood TBI. Their sample consisted of 16 pairs of former patients and parents after a mild, moderate, and severe pediatric TBI. The authors concluded that parents could act as potentially accurate substitutes for rating the psychosocial outcome [22]. These results are very consistent with the results of our study, although Green et al.'s participants were significantly younger at the time of injury and the TBI severity was heterogenic.

Most research on agreement between proxy-reporting and self-reporting systems has addressed adults. Hart et al. (2003) found agreement rates for neurobehavioral functioning between adult individuals with TBI and their SOs one year after injury to be moderately high. In the domains of aggression and depression, however, there was relatively low agreement between the proxy- and self-reporting systems [35].

The results of Dawson et al. regarding the community integration outcome showed a high level of agreement between proxy- and self-reports with respect to objective subscales, such as frequency of participation. However, a lower level of agreement between the reports was found for the more subjective scales, such as expectation and satisfaction with patient participation. Dawson et al.'s findings suggest that the use of proxy data for research purposes is acceptable, but a twofold assessment, including both patient and proxy evaluations, should be used to create goals and evaluate their outcomes [25].

Dawson et al. also reported lower proxy agreement for adult survivors who have sustained more severe TBIs [25]. This is in contrast to our findings where a sample of exclusively severe TBI patients produced an overall good agreement, compared to a sample of mixed severity (mild, moderate, and severe TBI victims) by Rosema et al. (2014) producing a lower level of agreement.

Overall, the high accordance showed that the perception of the psychological and behavioral functioning was very close in the patients and their SOs. Empirical findings supported an association between higher levels of self-awareness and better outcome [36]. However, an agreement demands not only the high self-awareness on the former patients' side, but also the ability for empathic and objective appraisement on SOs' side.

Not only have personal aspects contributed to the level of agreement, but it should also be noted that more specific survey items tend to elicit higher levels of agreement between patient and family member perceptions [21]. This might have contributed to our study's high level of agreement, since the ARS 18–59 and ABCL 18–59 mainly consist of very specific items.

### 5.3. Methodological Limitations

The single-center design is an obvious limitation of the present study, along with the relatively small sample size. Small sample sizes are a well-known and common problem in the TBI literature [22, 23, 37].

Since all former patients of the past 13 years who met the inclusion criteria were contacted, the time span after injury varies from one to 13 years. Participants with a longer time gap between the injury and the time of participation had more time to adjust to the new situation, define a new life, and receive more rehabilitation interventions. Hence, patients may perceive less psychological problems if interviewed at a more distant time since the original injury. On the other hand, a longer time since injury could also lead to more problems developing over time. However, after analyzing the data, no differences were found in the psychological outcome in relation to the time since injury, except in the categories of anxiety and depression, where higher scores were found in persons with longer time since injury.

The length of time to complete the follow-up survey might have influenced the response rate. The longer it takes to answer a survey, the more likely the response rate decreases. The ASR 18–59 and the ABCL 18–59 are quite complex and require a certain motivation and time effort for the former patients and their SOs to complete the survey. This could have led some participants to not complete the material. However, the returned questionnaires were filled out in a thorough manner, showing a very low rate of missing values. This fact supports the choice of the instruments for this study.

A further limitation was that no Danish norms for the measures existed, and therefore a comparison with the Danish population was not possible. North American norms were used for the ASR-18–59, with cognizance of existing cultural differences and the different welfare systems. However, the common procedure when using the ASEBA material is to use North American norms.

## 6. Clinical Implications and Future Studies

Despite the abovementioned limitations, the findings have important clinical implications. In order to support former patients in the chronic phase, it is of prominent importance to fully evaluate individuals' problems and impairments. Objective ratings assessing a patient's scope of psychological functioning are necessary. As mentioned, overt areas of psychological functioning demonstrated high consensus between self-rating and proxy-rating reports, thus indicating that either assessment could be used in isolation. Nonovert areas showed lower consensus, suggesting the importance of considering both self-rating and proxy-rating scores when assessing patient difficulties. Finally, regarding a long-term view, it is of prominent importance to obtain a thorough appraisal of the patients' psychological well-being. Ownsworth and Clare (2006) stated that greater awareness of deficits is associated with better treatment outcomes [36].

Future research should be performed on larger samples and with a repeating measure design to observe changes in agreement over time. More research is also needed to investigate factors that may influence agreement levels. By continuing to understand how factors influence psychological assessment systems, professionals will be able to better choose whether a self-rating or proxy-rating system would be more appropriate for the condition being evaluated. Future studies could also explore and evaluate the effects of using a dyadic perspective throughout rehabilitation. Reaching a high level of agreement could be used as a rehabilitation goal of patients and their families.

## 7. Conclusion

One purpose of this study was to evaluate the level of agreement between self-rated psychological outcome scores of former adolescent TBI patients, compared to their corresponding SOs' proxy-rating scores. Our findings suggest that the exclusive use of either a self-rating or a proxy-rating system would be appropriate for evaluating overt domains of psychological functioning. However, in the nonovert domains of psychological functioning, it may be beneficial to obtain both a self-rating score from the patient and a proxy-rating score from a significant other. The combination of the self-rating and proxy-rating scores for the nonovert domains may provide additional information to build a more complete objective assessment. Our results also suggest that extended follow-up after TBI is a positive way to support former patients and their significant others as they struggle with psychological difficulties.

## Figures and Tables

**Table 1:** Sample characteristics (*n* = 20).

Characteristics of patients	
Gender *n* (%)	
Male	16 (80%)
Female	4 (20%)
Age at injury, year, mean (SD)	18.1 (1.9) Range 6 (min 15, max 21)
Age at participation, year, mean (SD)	23.8 (4.9) Range 13 (min 18, max 31)
Time since injury, month, mean (SD)	66.1 (46.6) Range 145 (min 5, max 150)
Duration of PTA, days, mean (SD)	49.4 (34.8) Range 120 (min 10, max 130)
Length of stay, days, mean (SD)	70.5 (44.8) Range 185 (min 21, max 206)

SD: standard deviation; GCS: Glasgow Coma Scale; PTA: posttraumatic amnesia.

**Table 2:** Status of patient and significant others (*n* = 20).

Characteristics		*n* (%)
Relationship toward patient	Parents	18 (90.0)
	Caregiver	1 (5.0)
	Spouse	1 (5.0)

Cohabitation status	Living at home	17 (85.0)
	Living with partner	1 (5.0)
	Shared accommodation	1 (5.0)
	Living in nursing home	1 (5.0)

**Table 3:** Comparison of self-rating and proxy-rating with the normative sample.

Scale	Characteristics	Patient Mean *t*-score (SD) *n* = 36	Difference *D* with the mean *t*-score of the normative sample	*p* value	Significant other Mean *t*-score (SD) *n* = 20	Difference *D* with the mean *t*-score of the normative sample	*p* value
Syndrome scales	Anxiety and depression	57.03 (9.36)	2.68	0.095	55.00 (5.62)	0.65	0.611
	Withdrawal	58.58 (10.23)	4.48	0.013^*∗*^	55.00 (6.95)	0.90	0.569
	Somatic complaints	56.53 (7.69)	2.43	0.066	55.50 (5.41)	1.40	0.261
	Thought problems	54.64 (8.44)	0.49	0.730	54.40 (5.74)	0.25	0.848
	Attention problems	58.17 (8.46)	3.97	0.008^*∗∗*^	57.55 (4.99)	3.35	0.007^*∗∗*^
	Aggressive behavior	54.22 (6.25)	0.12	0.907	54.15 (4.67)	0.50	0.962
	Rule-breaking behavior	54.75 (6.15)	0.60	0.562	54.20 (4.05)	0.50	0.957
	Intrusive behavior	52.50 (4.65)	1.60	0.046^*∗*^	53.15 (5.52)	0.95	0.451

Combined scales	Externalizing	49.33 (10.07)	0.72	0.672	52.25 (9.82)	2.05	0.362
	Internalizing	54.83 (12.36)	4.63	0.031^*∗*^	50.90 (7.45)	0.85	0.616
	Total problems	48.90 (9.24)	1.20	0.568	50.65 (8.36)	0.55	0.772
	Critical items	55.50 (6.05)	1.40	0.314	55.85 (5.28)	1.75	0.155

SD: standard deviation; ^*∗*^significant = *p* < 0.05;  ^*∗∗*^significant = *p* < 0.01.

**Table 4:** Comparison of self-rating and proxy-rating.

Scale	Characteristics	Patient mean *t*-score (SD) *n* = 20	Significant other mean *t*-score (SD) *n* = 20	Difference between the mean two-tailed *p* values	Intraclass correlation (ICC)	*p* value of ICC	Level of clinical significance
Syndrome scales	Anxiety and depression	55.40 (6.73)	55.00 (5.62)	0.762	0.573	0.003	Fair
	Withdrawal	56.60 (8.57)	55.00 (6.95)	0.376	0.492	0.010	Fair
	Somatic complaints	55.55 (5.35)	55.50 (5.41)	0.948	0.813	<0.001	Excellent
	Thought problems	53.70 (6.34)	54.40 (5.74)	0.654	0.370	0.046	Poor
	Attention problems	56.80 (5.60)	57.55 (4.99)	0.541	0.495	0.010	Fair
	Aggressive behavior	53.45 (5.57)	54.15 (4.67)	0.423	0.727	<0.001	Good
	Rule-breaking behavior	53.10 (3.99)	54.20 (4.05)	0.248	0.464	0.015	Fair
	Intrusive behavior	52.35 (4.34)	53.15 (5.52)	0.405	0.646	0.001	Good

Combined scales	Internalizing	52.90 (10.80)	52.25 (9.82)	0.756	0.617	0.001	Good
	Externalizing	48.15 (8.62)	50.90 (7.45)	0.149	0.461	0.016	Fair
	Total problems	48.90 (9.24)	50.65 (8.36)	0.367	0.541	0.005	Fair
	Critical items	55.50 (6.05)	55.85 (5.28)	0.759	0.624	0.001	Good
	Personal strength	46.35 (10.81)	46.55 (7.20)	0.941	0.103	0.326	Poor

SD: standard deviation.

**Table 5:** Comparison between responder and nonresponders (contacted sample *n* = 80).

Patients characteristic	Mean (SD)	*p* value
*PTA (in days)*		
Responding (*n* = 35)	42.8 (29.3)	0.459
Nonresponding (*n* = 44)	43.8 (40.0)	
*Age at injury (in years)*		
Responding (*n* = 36)	18.0 (1.8)	0.441
Nonresponding (*n* = 44)	17.7 (2.1)	
*Age at participation (in years)*		
Responding (*n* = 36)	24.1 (4.1)	0.806
Nonresponding (*n* = 44)	24.3 (4.3)	
*Gender*		
Responding (*n* = 36)	Male 27 (75%)	
	Female 9 (25%)	
Nonresponding (*n* = 44)	Male 36 (79.5%)	
	Female 9 (20.5%)	

PTA: posttraumatic amnesia; SD: standard deviation.
